# Design of Automatic Extraction Algorithm of Knowledge Points for MOOCs

**DOI:** 10.1155/2015/123028

**Published:** 2015-09-13

**Authors:** Haijian Chen, Dongmei Han, Yonghui Dai, Lina Zhao

**Affiliations:** ^1^School of Information Management and Engineering, Shanghai University of Finance and Economics, 777 Guoding Road, Shanghai 200433, China; ^2^School of Open Education, Shanghai Open University, 288 GuoShun Road, Shanghai 200433, China; ^3^Shanghai Financial Information Technology Key Research Laboratory, 777 Guoding Road, Shanghai 200433, China; ^4^School of Information Management, Shanghai Finance University, 995 Shangchuan Road, Shanghai 200433, China

## Abstract

In recent years, Massive Open Online Courses (MOOCs) are very popular among college students and have a powerful impact on academic institutions. In the MOOCs environment, knowledge discovery and knowledge sharing are very important, which currently are often achieved by ontology techniques. In building ontology, automatic extraction technology is crucial. Because the general methods of text mining algorithm do not have obvious effect on online course, we designed automatic extracting course knowledge points (AECKP) algorithm for online course. It includes document classification, Chinese word segmentation, and POS tagging for each document. Vector Space Model (VSM) is used to calculate similarity and design the weight to optimize the TF-IDF algorithm output values, and the higher scores will be selected as knowledge points. Course documents of “C programming language” are selected for the experiment in this study. The results show that the proposed approach can achieve satisfactory accuracy rate and recall rate.

## 1. Introduction

Massive Open Online Courses (MOOCs) have played a great role in the process of construction of learning society [1]. With a rapid development of more than ten years of online learning, online learning resources have been seriously overloaded, and it is difficult for a learner to find suitable learning resources for his own learning resources [2]. Therefore, how to realize the knowledge sharing and knowledge discovery in MOOCs era has attracted the attention of experts in the field of education. The ontology technology is one of the effective ways to solve the knowledge sharing and knowledge discovery, more and more scholars apply it to MOOCs in recent years, and ontology construction has become a hot spot research. At present, most of the construction of domain ontology has to be done manually, which is using a plain document editor or ontology editing tools (such as protégé, Swoop, Ontolingua, and OntoEdit) to add one by one manually. Protégé is a very popular and useful tool [3, 4]. Obviously, this method is not only time-consuming, error prone and difficult to update, but also it needs the participation of experts in the field. The most important aspect is that the manual construction of ontology is inefficient, and it is hard to be popularized. Ontology learning usually use ontology engineering, machine learning technology, statistics and principles of many other subjects to realize the automatically or semiautomatically construction of ontology [5]. By ontology learning, concepts and classifications can be extracted from a variety of nonstructured document [6]. Automatic construction of ontology will greatly improve the development process of semantic ontology and easy to achieve knowledge discovery and knowledge sharing. It provides the possibility of course ontology reasoning and the necessary condition for personalized learning. In education domain, knowledge point is the basic elements and the foundation of the relationship between them. Hence, automatic extraction of knowledge is the key of ontology learning [7].

Generally, there are three ways for automatic extraction of knowledge in the field of education: linguistics method, statistical method, and hybrid method [8]. There are the following several advantages of Linguistic method high accuracy, small amount of calculation, without relying on the corpus, ability to extract low frequency point of knowledge, but with poor portability, it is difficult to maintain the rules of language. Even not relying on syntactic and semantic knowledge base and ability to process incomplete sentences or phrases properly without the restriction of different language, statistical method bear the disadvantage of huge calculation and difficulty to extract multimeaning knowledge points and low frequency knowledge points. Hybrid method is combining Statistics knowledge with linguistic knowledge (syntactic and semantic information), taking the advantage of both methods [9]. Considering the particularity of online course, we use the hybrid method, using linguistic methods to process Chinese word segmentation and POS tagging, and using statistics method to handle score method for characteristics.

In order to construct the educational domain ontology automatically, automatic extraction of knowledge point is a very important job. First, it classifies the document and then makes Chinese word segmentation and POS tagging for each document, it uses vector space model (VSM) to calculate similarity and design the weight value to optimize the TF-IDF algorithm value as the score for each feature value, and then sequence these characteristics by rating sort. Finally, the higher scores are selected as knowledge points. The experiment results show that the automatic extraction for knowledge has high accuracy rate and high recalling rate, lay a solid foundation for future automatic construction of course ontology.

This paper is arranged as the following seven sections.  Section 1 is the introduction of research background; Section 2 is related literature review; Section 3 expounds the methodology and technology as well as the TF-IDF algorithm, similarity calculation, and normalization method; Section 4 discusses the modeling and designing frameworks of automatic extracting course knowledge point; Section 5 illustrates the process and algorithm systematically; Section 6 is about the empirical analysis of “c programming language” course documents; and the conclusion and discussion are expressed in Section 7.

## 2. Related Researches

**Figure 1:**
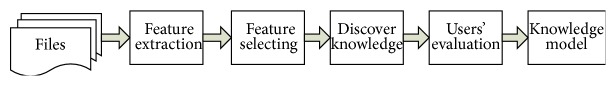
The general process of text mining.

The sorting of information in text resource cannot be realized without the text mining technology.  Figure 1 is the typical chart for the flow of text mining.

From Figure 1, it can be seen that the first step is to extract appropriate features from the text, which make the text into digital form that the computer can understand. According to the need for processing speed and accuracy, the features in text can be selected and optimized. Then, a variety of text mining methods will be used to discover the hidden knowledge patterns, the final output which meets the user's evaluation standard will be formed as useful knowledge to guide people's practice [10]. The essence of text mining is about text classification and feature extraction technology. The development of text classification has experienced two stages which are rule-based system and machine learning. Since 2000, the machine learning method has been widely used in text classification, when several training samples with manual annotation categories are designed, the system of machine-based learning can construct automatically text classification model, which improve the efficiency and performance of the classification. But no matter in which stage of text classification, expert's knowledge in the field plays a very important role; for example, the training samples should be labeled manually when using the classification method based on machine learning [11]. Therefore, in the design of text classification process, experts' knowledge in that field is taken as an important part of the system.

Generally, teaching document is semistructured or unstructured data; the knowledge point can be extracted automatically by using text mining. Research in other countries is mature and has proposed many fruitful methods, which is based on the study of English language. Missikoff's approach to ontology engineering uses an iterative process that involves automatic concept learning with OntoLearn [12]. Navigli et al. used it to automatically translate multiword terms from English to Italian [13]. Text mining produces a more structured analysis of textual knowledge than simple word searches and can provide powerful tools [14–16]. A personalized ontology model is proposed for knowledge representation and reasoning over user profiles [17]. As there is big difference between English language and Chinese language, there are fewer researches in the field of automatic extraction for Chinese language in China. Du et al. proposed a term extraction algorithm combining statistics-based method and rule-based method [18]. Zheng and Lu proposed a method that combined nonlinear function and “paired comparison method,” considered the location and frequency of words, gave the weight of candidate word, and realized the automatic extraction of keywords [19]. Chen et al. proposed automatic acquisition of field words from a large unlabeled corpus by using Bootstrapping machine learning technology [20]. Liu proposed methods which extract automatically webontLearn in the web pages [21]. In his study, He studied the relationship between semantic concepts from the data in the web page and how to extract automatically web ontology through the analysis of the same application field of web page set.

In the concept extraction, statistical method is mainly adopted, which is also the current mainstream technology. Rules-based approach is also applied to solve the key difficulty in field correlation of concept. By calculating the ratio between the frequency of the concept in the documents of particular field and frequency of the concept in the normal documents, correlation of the concept can be determined. That is, if the ratio is greater than the specified threshold, it means that the concept often appears in that particular field and is not often used in other fields.

## 3. Methodology and Technology

### 3.1. Concept Filters

Domain concept emerged in the field of corpus more frequently than it appeared in the General Corpus. If a concept appears in the field of corpus more frequently than it appears in the general corpus, it is considered related to the field [22, 23]. The concept of the area has the following two characteristics.

1. The words appear in the field more frequently than in other areas.
2. The concept in the field is commonly recognized, it is therefore widely used in the field.

These two characteristics can be measured, respectively, by the concept of Domain Relevant and Domain Consensus [24].

#### 3.1.1. Domain Relevant

The domain relevance of a concept *t* in domain *D*_*i*_ is given as follows:

(1) $$\begin{array}{c} DR\left(\;t,D_{i}\;\right)=\frac{p\left(t\;|\;D_{i}\right)}{\max p\left(t\;|\;D_{j}\right)}, \end{array}$$

where DR is in [0, 1]. According to the large number theorem of probability theory that, under the premise that large sample has the same base, the sample's frequency is close to the probability value, so the maximum likelihood estimation value of the conditional probability *p*(*t*, ∣*D*_*i*_) is equal to the frequency of “*t*” appearing in the field of *D*_*i*_, there is an equation that

(2) $$\begin{array}{c} p\left(\;t\;|\;D_{i}\;\right)=\frac{freq\left(t\in D_{i}\right)}{\sum_{i=1}^{n}freq\left(t\in D_{i}\right)}. \end{array}$$

#### 3.1.2. Domain Consensus

The domain consensus of a concept “*t*” in domain *D*_*i*_ is given as follows:

(3) DCt,DiHpt,dj=∑pt,dj×log2⁡1pt,dj,

where *d*_*j*_ is documents in *D*_*i*_, and the probability *p*(*t*, *d*_*j*_) is estimated as follows:

(4) $$\begin{array}{c} \frac{freq\left(t\in d_{j}\right)}{\sum_{d_{j}\in D_{i}}^{}freq\left(t\in d_{j}\right)}. \end{array}$$

#### 3.1.3. Concept Filters

After Qualify concept's Domain Relevant and Domain Consensus, the degree of importance for each candidate concepts “*T*” to domain *D*_*i*_ can be defined as follows:

(5) $$\begin{array}{c} CF\left(\;T,D_{i}\;\right)=\alpha \times DR\left(\;T,D_{i}\;\right)+\beta \times DC\left(\;T,D_{i}\;\right). \end{array}$$

In the above equation, *α*, *β* ∈ [0,1].

### 3.2. TF-IDF

Term Frequency-Inverse Document Frequency is a numerical statistic that is intended to reflect how important a word is to a document in a collection. It is often used as a weighting factor in information retrieval and text mining. The importance of a word is highlighted with the increasing of the times of its appearing in a file, but the importance is decreased inversely as the frequency of its appearing in the corpus. If a word or phrase bears high frequency in an article while with very low frequency in other articles, the word or phrase is usually taken as keyword with ability for distinguishing.

#### 3.2.1. Calculate TF

TF represents the number of a word appears in the document. Because documents have different lengths, the TF standardization is used to facilitate the comparison of different documents:

(6) TF=The  number  of  a  word  appears  in  the  documentThe  total  number  of  words  in  the  document.

#### 3.2.2. Calculate IDF

IDF is a measure of the importance of a common word. IDF's main idea is as follows: if the document contains fewer entries, IDF becomes bigger; the entry bears the ability to distinguish between good categories.

(7) IDF=log⁡Total number of documents in corpusTotal number of documents containing the term+1.

#### 3.2.3. Calculate TF-IDF

TF and IDF together can form TF-IDF measure:

(8) $$\begin{array}{c} \text{TF-IDF}=TF\times IDF. \end{array}$$

As you can see, the value of TF-IDF is directly proportional to the frequency of a word's appearing in the file, but inversely proportional to the frequency of the word's appearing in the entire corpus.

### 3.3. Similarity Algorithm

Each word *W* is considered as a vector:

(9) $$\begin{array}{c} W=\left[\;w_{1},w_{2},w_{3},\ldots ,w_{n}\;\right]. \end{array}$$

A lot of similarity algorithms have been proposed and widely applied on similarity calculation, such as cosine similarity, Jaccard coefficient, and Pearson Correlation Coefficients. The details of different similarity measures are described as below.

*(i) Cosine Similarity.* Cosine similarity is a measure of similarity between two vectors, which measures the cosine of the angle between them [25]. The cosine of 0° is 1, and it is less than 1 for any other angle. Compared to the distance measure, the cosine similarity pays more attention to the differences between the two vectors in the direction, rather than the distance or length. The formula is as follows:

(10) $$\begin{array}{c} sim\left(\;w_{j},w_{i}\;\right)=\cos\theta =\frac{w_{i}\cdot w_{j}}{\left\|w_{i}\right\|\cdot \left\|w_{j}\right\|}. \end{array}$$

*(ii) Jaccard Coefficient.* The Jaccard coefficient measures similarity as the intersection divided by the union of the objects. The Jaccard coefficient is mainly used for computing symbol metric or Boolean similarity between individual attributes, because the individual is symbol metric or a Boolean indicator therefore unable to measure the difference of specific value, can only get “is the same as” the results, the Jaccard coefficient is concerned only with the common features among individuals is consistent with this problem [26]. The formula is as follows:

(11) $$\begin{array}{c} sim\left(\;w_{i},w_{j}\;\right)=\frac{w_{i}\cap w_{j}}{w_{i}\cup w_{j}}. \end{array}$$

The Jaccard Coefficient ranges between [0, 1]. The Cosine Similarity may be extended to yield Jaccard Coefficient in case of Binary attributes. 

*(iii) Pearson Correlation Coefficients.* In statistics, Pearson correlation coefficient is used to measure the relationship between the two variables *X* and *Y* (linear), in the range [−1, +1]. Pearson correlation coefficient is widely used in academic research to measure the two variable linear correlations [27]. The formula is as follows:

(12) $$\begin{array}{c} sim\left(\;w_{i},w_{j}\;\right)=\frac{Cov\left(w_{i},w_{j}\right)}{\sqrt{Var\left(w_{i}\right)\cdot Var\left(w_{j}\right)}}. \end{array}$$

Cov(*w*_*i*_, *w*_*j*_) represent the covariance of *w*_*i*_ and *w*_*j*_, Var(*W*_*i*_) represent the variance of *w*_*i*_, and Var(*W*_*j*_) represent the variance of *w*_*j*_.

### 3.4. Normalization Method

Normalization method is a basic task of data mining; different evaluation index often have different dimension and dimensional units; this situation will affect the results of data analysis. In order to eliminate the dimensional effects between the indexes, normalization method is frequently used. After data standardization processing which is each index of the original data at the same level, suitable for evaluation of comprehensive comparison. The data is mapped to [0, 1] interval method for data normalization includes: Min-Max normalization, log function, atan function, and zero-mean normalization. We use Min-Max normalization in this paper; the formula is as follows:

(13) $$\begin{array}{c} x^{*}=\frac{x-min}{max-min}. \end{array}$$

## 4. Frameworks and Processes

**Figure 2:**
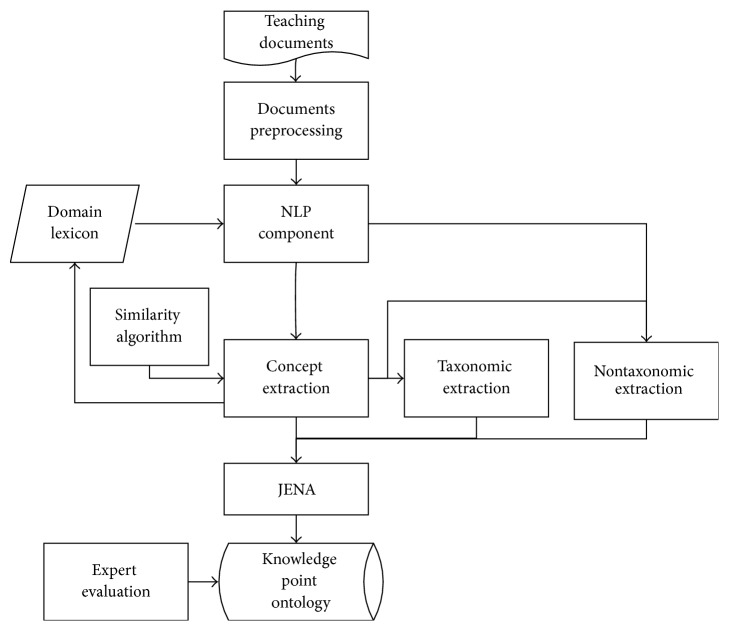
The frameworks of automatic knowledge points' extraction.

There is great difference between the extraction for course knowledge point and the extraction for general feature in common document. The extraction for general feature is to study and analyze mass documents and find out the feature value which can represent a field, commonly used in document classification, document clustering, information extraction, relation analysis, and so on. The following are the methods for feature extraction (evaluation): document frequency (referred to as DF), information gain (referred to as IG), mutual information (referred to as MI), expected cross entropy, the weight of evidence for document, odds ratio, and so on. The experimental results show that DF and IG result well [28]. There are a lot of researches on the feature selection. Yang et al. and Feng et al. pointed out that extraction of curriculum knowledge is to extract knowledge automatically from the curriculum teaching files, teaching content, database, and other documents by using Chinese segmentation and text mining techniques, that is to structure or semantic the unstructured documents for the follow-up research work of knowledge sharing and knowledge discovery [29, 30]. Because it is in a specific environment and there is a strong correlation between document and knowledge points in the online course, so using VSM model will greatly reduce the feature dimension. At the same time, by increasing the “knowledge-Document” matrix design weight algorithm and optimizing the document frequency method, improve the extraction effect for course knowledge point. Framework of automatic extraction for course knowledge points as shown in Figure 2.

The whole process consists of seven steps, as follows.

### 4.1. Documents Preprocessing

Curriculum resource of online course is rich; the content and style of the course are varied, they generally include teaching files, teaching content, exercises, case base, question library, video library and so on. The first step is to classify documents and taking the following three types of documents, which are very important in almost every course: the teaching files, teaching contents, and exercises. The teaching file is a programmatic document which has large and comprehensive contents; teaching contents include detail contents of each chapter; exercises is to measure teaching quality of this course. The above three documents contain all the knowledge points of a course. Secondly, considering the diversification of the types of the document which shows in PDF, HTML, XML, Excel, and other different formats, this document needs to be unified into a plain document file format (*∗*.txt) [31].

### 4.2. Chinese Word Segmentation and POS Tagging

Chinese language is read sentence by sentence, which is different from English word, so we need to perform segmentation on Chinese document. Chinese word segmentation is the process of dividing written text into meaningful units, such as Chinese words, Chinese sentences, or Chinese topics. Software ICTCLAS is used to divide sentences into words and tag words in this paper. Because dividing sentence into words belongs to the category of linguistics, different factors will lead to different results [32]. For example, “the foundation of program design” in Chinese idiom can be divided into “program,” “design,” and “the foundation of” or be divided into “program design” and “the foundation of” or be divided by other ways. Therefore, the dictionary should be referred to when the sentences were divided into words; a number of keywords in a field and corresponding frequency should be added into the dictionary. Considering the background of this study, the dictionary in education field, dictionary in computer science field, and dictionary in curriculum field should be composed.

### 4.3. Candidate Knowledge Point

To process the segmentation results, VSM model was used to calculate the characteristics of TF-IDF algorithm using the TF-IDF value, then candidate course knowledge points were obtained by sequencing. Because most of the knowledge points are names and verbs (a lot of knowledge is a verb, e.g., “cycle” is a very important knowledge, but in Chinese it refers to a verb), so to reduce the number of useless adjectives and adverbs, articles can greatly reduce the dimension and improve the time complexity degree for VSM model. Then, calculate their frequency and inverse-document frequency for each feature. Because the relations between knowledge points will be extracted, the property of each candidate course knowledge points should be contained, including the location of the document, the document size in bytes, the position of the paragraph, the sentence position and other candidate knowledge in the same sentence.

### 4.4. Similarity Calculation

Because there are couples of expression for a same knowledge point; for example, the “branch structure” in “C language program design” can also be called “conditional structure,” “single branch,” or “multi branch.” So the similarity-value of knowledge points needs to be calculated. The knowledge points bearing similar similarity-value can be merged.

### 4.5. Weight Calculation and Normalization

Use “knowledge-document” matrix to calculate the weight of candidate knowledge points. Because all the documents are from the online course, there is strong relationship between knowledge and document. Considering the special nature of teaching content document and exercises for each chapter, “knowledge-document” matrix can be built to calculate the weight of each knowledge point, and then the weights are normalized.

### 4.6. Extraction for Knowledge Point

The frequency and correlation of candidate knowledge points are used to analyze weight and knowledge entropy weight and recalculate the frequency of candidate knowledge points. Then, course knowledge points are selected according to the sequence of the above calculating results.

### 4.7. Expert Evaluation

Experts determine knowledge point according to the characteristic of the curriculum field then compare to them by the knowledge points extracted automatically and analyze the reasons for the difference.

## 5. Algorithm Design

It is considered that online courses have distinctive feature; Automatically Extract Course Knowledge Points (AECKP) are designed in this paper to extract a certain course knowledge points automatically which includes the TF-IDF, similarity, weight algorithm, and the improved TF-IDF algorithms.

### 5.1. TF-IDF Calculation

The key point of TF-IDF (term frequency-inverse document frequency) is that if a knowledge point has high frequency in particular documents while seldom appears in other types of documents, this kind of knowledge point bears high capacity to distinguish category, thus has high degree of importance [33].

TF (Term Frequency) refers to the frequency a word appears in a document. Equation (14) means the frequency of kp (a knowledge point) in document *d*; kp_all_ means all the candidate knowledge points:

(14) $$\begin{array}{c} tf\left(\;kp,d\;\right)=\frac{count\left(kp,d\right)}{count\left(kp_{all},d\right)}. \end{array}$$

The main point of IDF Inverse Document includes the less the document which contains the knowledge point and the higher the IDF, which means the knowledge point is very important. Equation (15) represents the frequency of IDF in the whole documents collection, and *N* means the total number of documents in *D*_*i*_ document collection:

(15) $$\begin{array}{c} idf\left(\;kp\;\right)=\log\left(\;\frac{N}{docs\left(kp,D_{i}\right)+1}\;\right). \end{array}$$

Equation (16) is about TF-IDF model; it is to calculate the value of TF-IDF for each knowledge point according to tf and idf. *D*_*ij*_ means the document sequenced by *j* in *D*_*i*_ document collection, and *N* means the total numbers of documents in *D*_*i*_ document collection:

(16) $$\begin{array}{c} \text{tf-idf}\left(\;kp,D_{i}\;\right)=\overset{N}{\underset{j=1}{\sum }}tf\left(\;kp,D_{ij}\;\right)*idf\left(\;kp\;\right). \end{array}$$

While judging the importance of the documents, TF-IDF considers not only the frequency of a knowledge point in a document (word frequency) but also the IDF of the knowledge point in all kinds of documents.

### 5.2. Similarity Calculation

Extract the feature vector of two candidate knowledge points in any domain concept, respectively, and then calculates the semantic similarity between them using the cosine method. The equation can be as shown in

(17) $$\begin{array}{c} \cos\left(\;{KP}_{i},{KP}_{j}\;\right)=\frac{\sum_{i=1}^{k}X_{i}Y_{i}}{\sqrt{\sum_{i=1}^{k}X_{i}}\sqrt{\sum_{i=1}^{k}Y_{i}}}. \end{array}$$

In (17), KP_*i*_ and KP_*j*_ represent two knowledge points, *X*_*i*_ and *Y*_*i*_ represent the feature vector, and *K* represents the number of feature vector.

### 5.3. Weight Calculation and Normalization

The calculation of Document TF-IDF is for mass text mining; for this particular environment of online course, the effect is not ideal. This paper adopts “knowledge point-document” matrix to calculate the weight value of each knowledge point. According to the above classification, “knowledge point-teaching file,” “knowledge point-teaching content,” and “knowledge point-exercises” matrix were established. “Knowledge-teaching content” matrix is shown in Table 1.

Consider

(18) a1=maxi=1⋯M1⁡countkp,dicountkp,D1,a2=maxi=1⋯M2⁡countkp,dicountkp,D2,a3=maxi=1⋯M3⁡countkp,dicountkp,D3.

In (18), *a*_1_ represents the weights of knowledge point in the teaching file, *D*_1_ represents the teaching file collection, *M*_1_ represents the total number of teaching file collection, *a*_2_ represents the weight of knowledge point in teaching contents collection, *D*_2_ represents the teaching content collection, *M*_2_ represents the total number of teaching content collection, *a*_3_ represents the weight of knowledge point in exercise Library, *D*_3_ represents the exercises in the document collection, and *M*_3_ represents the total number of exercises in the document.

Min-Max normalization method is used to normalize the weight as shown in

(19) $$\begin{array}{c} a_{i}^{*}=\frac{a_{i}-\min\left(a_{i}\right)}{\max\left(a_{i}\right)-\min\left(a_{i}\right)}. \end{array}$$

### 5.4. Improved TF-IDF Algorithm

In this paper, the TF-IDF algorithm is added with weight to form the improved TF-IDF, named I-TF-IDF:

(20) $$\begin{array}{c} \text{I-TF-IDF}\left(\;kp\;\right)=\overset{3}{\underset{i=1}{\sum }}\alpha_{i}^{*}\times TF\left(\;kp,D_{i}\;\right)*IDF\left(\;kp\;\right). \end{array}$$

In (20), *D*_*i*_ represents the document collection numbered *i*, TF represents KP's frequency in *D*_*i*_, IDF represents KP's inverse document frequency in *D*_*i*_, and *α*_*i*_^*∗*^ indicates the normalization weight of document numbered *i*.

In this paper, the weighted word frequency values were calculated by I-TF-IDF algorithm, normalization, and sequencing. We choose 80 as the threshold value in 1st level of knowledge points and 200 as the threshold value in 2nd level of knowledge points; the knowledge point whose calculating results is greater than the threshold is taken as course knowledge point being extracted automatically.

## 6. Experiment

This experiment adopts C# language and SQL2005 to write program and uses SharpICTCLAS to make word segmentation and POS tagging. SharpICTCLAS is word segmentation system, which is provided by China Academy of Sciences.

**Figure 3:**
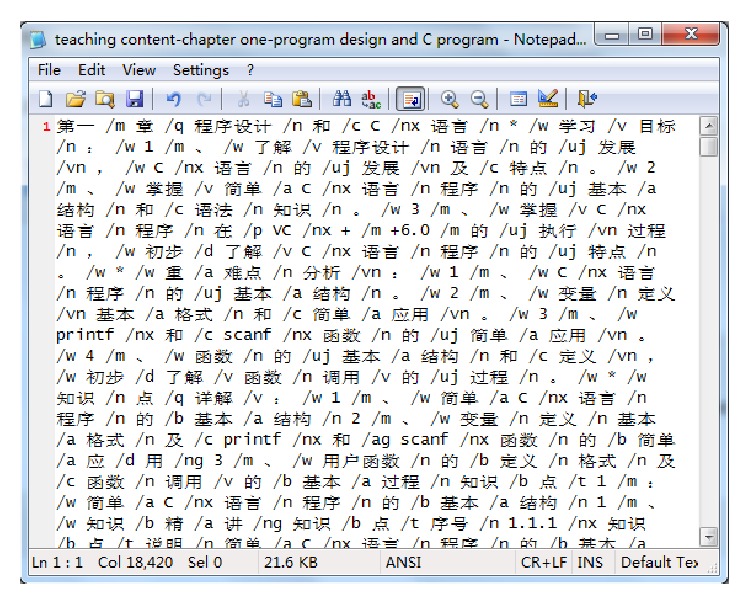
Result of word segmentation and POS tagging.

In this paper, “C programming language” was selected as the experiment, the 68 study documents about “c language” were downloaded in the MOOCs platforms from 8 colleges and universities. The results of word segmentation and POS tagging about “c programming language” document as shown in Figure 3.

Course knowledge points were extracted automatically by using the AECKP algorithm; the precision rate, the recall rate, and *F*_measures_ were analyzed and were compared with the knowledge point marked by experts [34]:

(21) precision=correctExpertsMark×100%,recall=correctall×100%,Fmeasures=2×precision×recallprecison+recall.

In (21), “correct” represents the number of correct knowledge points being extracted automatically, “all” represents the whole number of all knowledge points extracted automatically, “ExpertsMark” means the number of the knowledge point marked by experts.

Curriculum experts make hierarchical annotation for the knowledge in “C language program design,” divided the knowledge points into two levels. There are 66 knowledge points for the 1st level; there are 258 knowledge points for the 2nd level. There are 1953 candidate knowledge points extracted through AECKP algorithm, including 48 knowledge points in the first level and 193 knowledge points in the second level. The results of accuracy rate of two level knowledge points are shown in Table 2.

From Table 2, we can see that there is no close relation between the number of the knowledge points extracted by experts and the accuracy of knowledge points' extraction.

**Figure 4:**
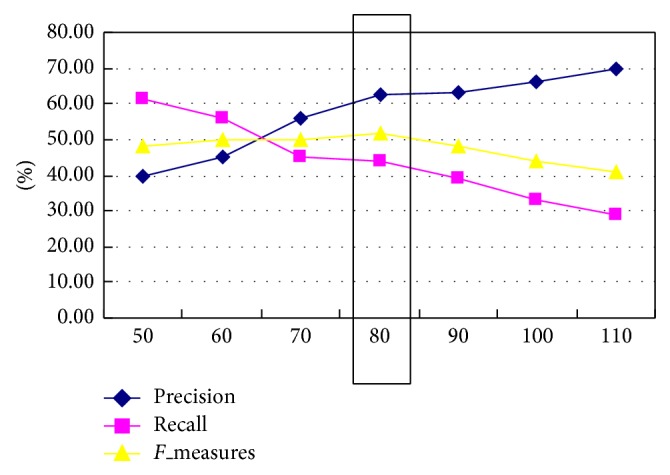
The best different threshold value in 1st level of knowledge points.

**Figure 5:**
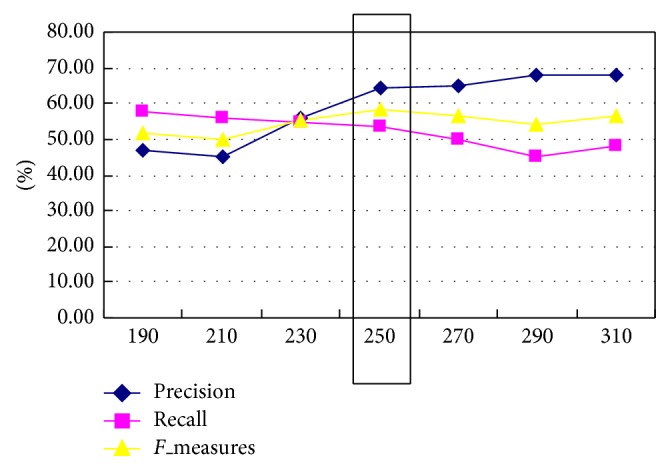
The best different threshold value in 2nd level of knowledge points.

In our experience, we choose different threshold in 1st level and 2nd of knowledge points, and the best different threshold values of them as shown in Figures 4 and 5.

From Figure 4, we can find 80 is the best threshold value. From Figure 5, it can be seen that 250 is the best threshold value. Then, the course knowledge points are greater than 80 in 1st knowledge points and greater than 250 in 2nd knowledge points are selected as the candidate knowledge points.

The study results are shown in Table 3.

From Table 3, it can be seen that once increase the number of expert annotation knowledge points, precision, recall and *F* test value will increase obviously. The main reason is that the number of candidate points did not change while the expert annotation knowledge increased in number, so the possibility of being relatively selected will increase. In addition, it can be seen from Table 3 that compared with TF-IDF algorithm, the accuracy and recall rate of AECKP algorithm on the course knowledge point extraction are improved to a certain extent, at the same time the extraction of low efficiency knowledge points is also improved.

**Figure 6:**
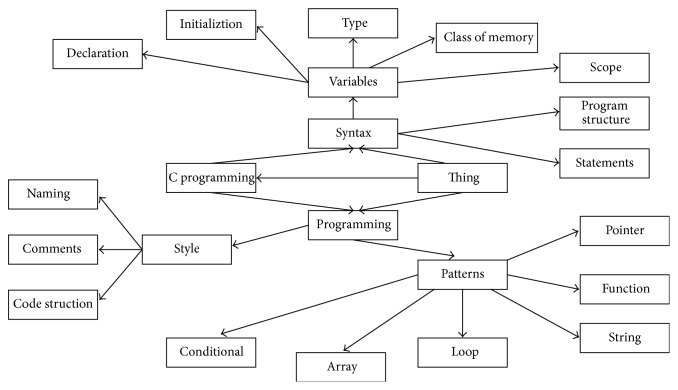
The partial educational ontology of C programming.

In our studies, we use the AECKP algorithm to extract the C language curriculum knowledge points and then use Jena to generate ontology automatically, the partial educational ontology of C Programming as shown in Figure 6.

## 7. Discussion

The necessity of automatic extraction for course knowledge points in ontology learning is analyzed, and the weakness of characteristics extraction algorithm which is usually used to extract common documents in online course is summarized in this paper.

Automatic ontology construction includes extracting ontological elements from input and building ontology from them [35]. It aims at building ontology from a given text corpus semiautomatically or automatically with a limited human exert. We usually define automatic ontology construction as a set of methods and techniques which are used to build ontology from scratch and use several sources in a semiautomatic fashion to enrich or to adapt to an existing ontology [36]. Automatic ontology construction uses methods from a diverse spectrum of fields, the field is varied from machine learning, knowledge acquisition, natural-language processing, information retrieval, artificial intelligence, and reasoning to database management [37, 38].

In addition, with the characteristics of education field considered, AECKP algorithm is proposed with details including algorithm frame, process, and algorithm design, and its performance is tested by experiment of which the results show high accuracy and recall rates. Due to the fact that the selected course “C language program design” contains both English and Chinese knowledge points, while the word segmentation module can only process Chinese words, therefore, English knowledge points are ignored during the statistical process.

Automatic extraction for course knowledge point is only a part of the course ontology learning. In future study, the relationship among knowledge points, including sequence relation and inclusion relation will be focused, extraction of relations among knowledge points automatically from the teaching document for automatic construction of course knowledge ontology will be studied to implement the ontology learning in a better way. Furthermore, the learners' interest as well as their possible emotional reactions may be considered as one of the features associated with the course knowledge points through the intelligent behavioral data-mining [39], speaker's recognition and affective computing on the vocal signals from learners' historical online study [40, 41].

## Figures and Tables

**Table 1:** “Knowledge point-teaching content” matrix.

Knowledge point	Teaching content 1	Teaching content 2	⋯	Teaching content m1
Constant	2	1	⋯	0
Variable	8	3	⋯	1
Integer	3	2	⋯	1
Float	1	1	⋯	0
Array	0	0	⋯	0
Function	0	0	⋯	0
Style	0	6		0
⋮	⋮	⋮	⋮	⋮

**Table 2:** The results of accuracy rate of two level knowledge points.

Parameters	The 1st level of knowledge points	The 2nd level of knowledge points
The expert annotation number of knowledge points	66	258
Extract expert annotation number of knowledge points	48	193
Accuracy rate	72.7%	74.8%

**Table 3:** The results contrast.

	The 1st level of		The 2nd level of	
Index	knowledge points		knowledge points	
	TF-IDF	AECKP	TF-IDF	AECKP
ExpertsMark	66	66	258	258
All	80	80	250	250
Correct	31	48	121	193
Precision	47.0%	72.7%	46.9%	74.8%
Recall	38.8%	60.0%	48.4%	77.2%
*F*_measures_	42.5%	65.7%	47.6%	76.0%
